# The Effect of Glucocorticoids on Angiogenesis in the Treatment of Solid Tumors

**Published:** 2020

**Authors:** Bing Liu, Julie E. Goodwin

**Affiliations:** 1 Department of Pediatrics, Yale University School of Medicine, New Haven CT 06520, USA; 2 Vascular Biology and Therapeutics Program, Yale University School of Medicine, New Haven CT 06520, USA

**Keywords:** Glucocorticoids, Angiogenesis, Malignancy

## Abstract

Glucocorticoids are steroid hormones produced by the adrenal cortex in a circadian manner and they participate in many physiological and pathological processes. Synthetic glucocorticoids have been universally applied to treat inflammatory diseases and immune disorders. Due to their angiostatic property, glucocorticoids are often added to regimens for cancer treatment. In the current review, we summarize how glucocorticoids influence angiogenesis in common solid tumors based on literature from the last ten years. Usage of glucocorticoids can be a double-edged sword in the treatment of some malignancies. There are still unanswered questions about the role of glucocorticoids in the treatment regimens of some common cancers. Therefore, we suggest prudent and restricted administration of glucocorticoids to treat solid tumors.

## Glucocorticoids and the Glucocorticoid Receptor.

Glucocorticoids (GCs) are defined by their role in maintaining glucose homeostasis and natural GCs are a class of corticosteroids secreted by the adrenal cortex [1]. Cortisol is the most important natural GC in humans. Cellular cortisol levels are regulated by the tissue-specific metabolic enzymes 11β-hydroxysteroid dehydrogenase 1 and 2 (11β-HSD 1 and 2); 11β-HSD 1 converts inactive cortisone to active cortisol, while 11β-HSD 2 has the opposite function [2]. The relative activity of these enzymes is responsible for maintaining the balance of cortisol *in vivo*. The release of cortisol into the circulation is involved in a variety of systemic processes such as immune responses, metabolism, cell growth, development, and reproduction [3]. Additionally, due to the multi-functional features of GCs, more and more synthetic GCs, such as hydrocortisone, dexamethasone (DEX), prednisone (PRED), triamcinolone acetonide (TA) and budesonide (BUD) are being widely-prescribed in clinical settings [4,5].

GCs trigger gene transcription by interacting with the glucocorticoid receptor (GR). GR is encoded by the gene NR3C1 and acts as a ligand-inducible transcription factor [6]. By specific alternative splicing, GR has several common isoforms, including GRα and GRβ. GRα is the classical GR isoform which has strong binding affinity for GCs, while the GRβ splice variant does not bind GCs, instead functioning as a natural inhibitor of the GRα isoform on many GC-responsive target genes [7]. Increased expression of GRβ has been associated with GC resistance, which may be due to competition for transcriptional co-regulators, or the formation of inactive GRα/GRβ heterodimers [2].

GC-GR signaling functions in two ways: via genomic effects and via non-genomic effects [8]. The former is the canonical manner which depends on GR-mediated transcription and protein synthesis. Once binding with GCs, GR rapidly translocates to the nucleus and subsequently regulates the transcription of its target genes through genomic mechanisms [9]. Unlike the genomic effect, which usually takes place in hours, the non-genomic effect of GC-GR signaling can arise within minutes because such an action is initiated at the cell surface via either membrane binding [10] or cytoplastic GR [11], rather than requiring GR to translocate to the nucleus. Due to different mechanisms between genomic and non-genomic effects of GC-GR signaling, GCs also exert distinct actions in different diseases.

### Mechanisms of GC regulation of angiogenesis

GCs are angiostatic and are used to treat angiogenesis-related diseases, including diabetic retinopathy and solid tumors. Several studies illustrate how GCs regulate angiogenesis. As shown by Shikatani et al., corticosterone decreased the number of capillaries in rat skeletal muscle and inhibited proliferation, migration, and sprouting of skeletal muscle microvascular endothelial cells *in vitro* [12]. After treatment with corticosterone, endothelial cells showed diminished VEGF mRNA levels, as well as reduced production and activation of MMP-2, correlating with inhibition of cell sprouting within a 3D collagen matrix. The expression of Sp1, a transcriptional regulator of both VEGF and MMP-2, was also demonstrated to be down-regulated by corticosterone. *In vitro*, TA treatment decreased the protein expression of the cellular and soluble forms of VEGF and VEGFR-1 as well as vascular network forming capacity through the Akt/mTOR pathway in a time- and dose-dependent manner [13].

**Figure 1:**
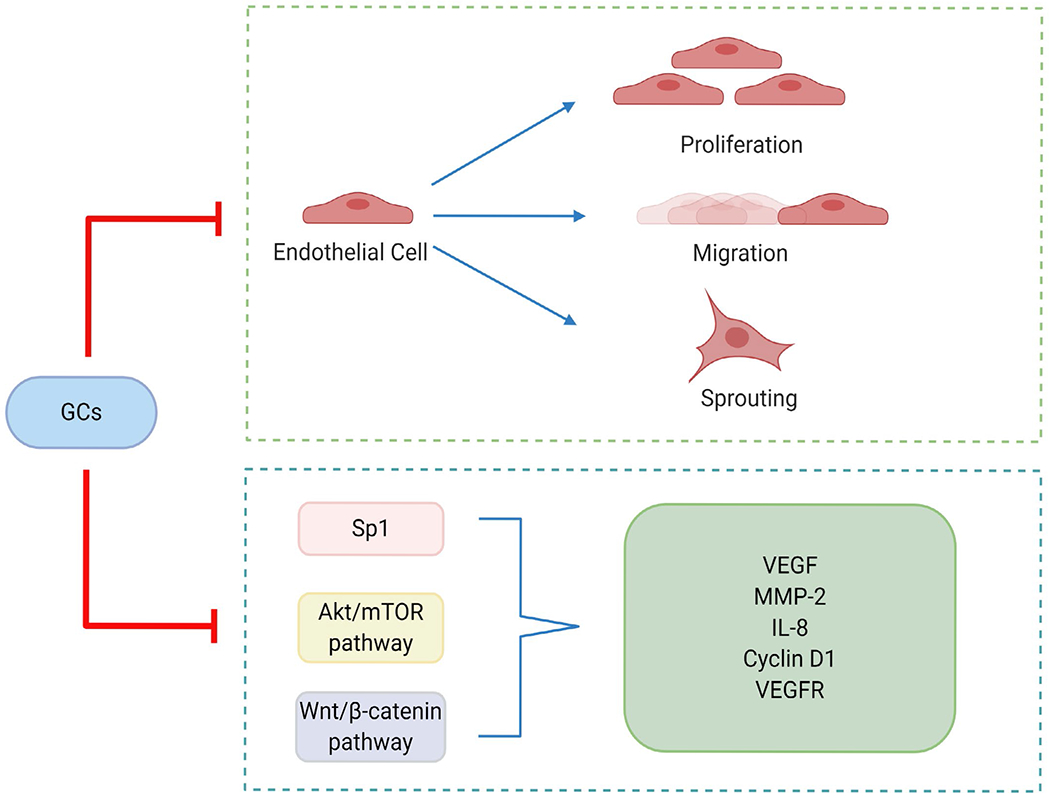
The regulatory effects of GCs on angiogenesis

The Wnt/β-catenin signaling pathway also appears to be critical in vascular endothelial cells and acts through a variety of regulators, including Bach1 [14], Rspo1 [15], Endostar [16], and ERG [17]. Once Wnt/β-catenin signaling is triggered, β-catenin binds to TCF in the nucleus, and alters the expression of key drivers and regulators of angiogenesis, such as VEGF, IL-8, Cyclin D1 and MMP-2 [18]. Using a ChIP-seq approach as well as a validate mouse model, we have recently shown that loss of endothelial GR results in the upregulation of Wnt signaling both *in vitro* and *in vivo* [19]. Therefore, we hypothesize that steroid microenvironments in endothelial cell networks have important implications for regulation of angiogenesis. The regulatory effects of GCs on angiogenesis are shown in Figure 1.

In general, GCs can regulate angiogenesis via two main approaches: (i) suppression of proliferation, migration and sprouting in endothelial cells and (ii) reduction of the secretion or expression of key cytokines and/or proteins responsible for the upregulation of angiogenesis.

### Role of GCs in Cancer Treatment

Synthetic GCs have been globally applied for the treatment of inflammatory and immune disorders, including rheumatoid arthritis, multiple sclerosis, inflammatory bowel disease, and nephrotic syndrome [20]. In addition, owing to their ability to induce apoptosis in hematological cells, GCs are used as chemotherapeutic agents for the treatment of acute lymphoblastic leukemia (ALL), chronic lymphoblastic leukemia (CLL), multiple myeloma (MM), Hodgkin’s lymphoma (HL) and non-Hodgkin’s lymphoma (NHL) [21–25].

For non-hematologic malignancies, GCs can have either adjuvant or curative effects, depending on the subtype of tumor as well as the specific treatment protocols. For instance, due to their anti-emetic and anti-edemic properties, GC administration is nearly always added to surgery, radiotherapy or chemotherapy, where they can relieve symptoms of the primary disease, alleviate side effects of chemotherapy, and protect healthy tissues from cytotoxic effects induced by chemotherapeutic treatment [26–28].

In breast cancer, data from an animal model [29] demonstrated the treatment with TA decreased capsular thickness of the tumor, mild mononuclear inflammation, and negative or minimal angiogenesis in rabbits. However, according to Flaherty’s study, both in 66CL4 breast cancer cells and the mouse breast cancer model, GCs can induce DNA damage through an inducible nitric oxide synthase (iNOS)- mediated pathway by increasing levels of nitric oxide (NO); increased NO further stimulated by GC signaling may serve to promote angiogenesis through VEGF in a chronic stress model [30].

In prostate cancer, Yano et al. revealed that GCs acted directly through GR and suppressed two major angiogenic factors, VEGF and IL-8, in the androgen-independent prostate cancer cell line DU145. Additionally, in a xenograft model, except for intratumor VEGF and IL-8 gene expression, DEX treatment also inhibited angiogenesis and *in vivo* tumor growth [31]. Nevertheless, evidence exists that the GC signaling pathway can increase the diameter of blood vessels and vessel area in tumor tissues from prostate cancer patients [32].

In bladder cancer, Ishiguro et al. [33] showed that both DEX and PRED could repress the expression of MMP-9, VEGF, and IL-6 in UMUC3 and TCC-SUP human urothelial carcinoma cell lines. However, another study evaluated the effects of DEX on cell proliferation, apoptosis, and invasion in bladder cancer cells lines and found that, although DEX impeded cell invasion and the expression of angiogenesis-related genes (MMP-2/MMP-9, IL-6, and VEGF), as well as induced mesenchymal-to-epithelial transition, it also correlated positively with cell proliferation in mouse xenograft models and resulted in a significant reduction in the curative effects of cisplatin [34].

In glioblastoma multiforme (GBM), DEX treatment didn’t result in any changes either in total vessel area or average vessel size compared to vehicle treatment in a mouse model. Furthermore, clinical data implied that GCs might decrease the effectiveness of radiotherapy and chemotherapy as well as reduce overall survival in GBM patients [35]. According to Llaguno-Munive’s study [36], mifepristone (Mife), which is considered an antiglucocorticoid, was used in combination with chemoradiotherapy (Rad) and Temozolomide (Tmz) to treat GBM in mice. After 25 days, the tumor volume of the Rad + Tmz + Mife group was significantly less than that of the Rad + Tmz group. Furthermore, the expression of VEGF also decreased in the Rad + Tmz + Mife group. Several other clinical studies also showed that a decrease in GC use could improve the prognosis of GBM [37,38].

In melanoma, a new class of cationic lipid-DEX conjugate containing a C-8 carbon chain analogue (DX8), was used to study the efficiency of GCs on tumor-bearing mice. After calculating the area stained by VEGFR2 in endothelial cells, DX8 decreased the level of VEGFR2 in tumor-endothelial cells, implying DX8’s anti-angiogenic role in melanoma [39]. As demonstrated in Licarete’s study [40], the administration of prednisolone disodium phosphate (PLP) could improve doxorubicin cytotoxicity on B16.F10 murine melanoma cells *in vitro* via the inhibition of the proangiogenic function of tumor-associated macrophages (TAMs). Another study [41] revealed that HYC16, a kind of cationic lipid modification of hydrocortisone, exhibited significantly less VEGFR2 expression and lower density of vascular endothelial cells in mice, indicating HYC16 had an evident anti-angiogenic effect and substantiated its ability to inhibit tumor growth.

In HCT116 and HT29 colon cancer cell lines, DEX treatment inhibited HIF-1α protein levels and its downstream gene, VEGF mRNA levels. Also, the presence of DEX suppressed the mRNA levels of hypoxia-induced Snail, Slug, and Twist as well as hypoxia-induced integrin αVβ6 protein levels, which is a well-known EMT marker for colon cancer cells [42]. Based on Patras’s study [43], prednisolone-loaded long-circulating liposomes (LCL-PLP) + LCL-5-FU combination therapy resulted in lower expression of M-CSF, MCP-t, eotaxin, leptin, G-CSF, IGF-II, IL-1α, IL-1β, IL-9, IL-12p40, FasL, bFGF, and VEGF in C26 colon carcinoma tissue, which implies antiinflammatory and anti-angiogenic effects of LCL-PLP.

In a rat liver cancer model, compared to a glucuronolactone alone group, tumor nodule number and micro vessel density in the glucuronolactone + hydrocortisone group were significantly lower at week 12. Additionally, significantly decreased levels of macrophages, TNF-α, p-p38, NF-κB, IL-10, HGF, TGF-β1, and VEGF were observed in the paraneoplastic tissue of the glucuronolactone + hydrocortisone group when compared with the glucuronolactone group. The results suggest that hydrocortisone treatment reduces macrophage polarization, inflammatory and antiinflammatory cytokines levels, and angiogenesis in paraneoplastic tissue [44]. Similar results were also obtained in a mice model as the tumor weight in the DEX treatment group was observed to be significantly lower than that in the control group. Both tumor blood vessel density and total blood vessel length in the DEX group were smaller than those in the control group. These results indicate that DEX has an inhibitive effect on tumor growth and angiogenesis in murine liver cancer *in situ* [45].

As reported in Geng’s study [46], Lewis lung carcinoma cells were inoculated in C57BL/6 mice, and the mice were randomly divided into 3 groups: a control group, a cisplatin group, and a DEX group. The results demonstrated that tumor growth was suppressed in the both cisplatin group and the DEX groups. In addition, tumor weights decreased in the cisplatin and DEX groups compared to the control group. The expression of HIF-1α and VEGF and the density of micro vessels were also significantly lower in the cisplatin and DEX groups than in the control group. However, these changes were not significantly different between the cisplatin group and DEX group, indicating DEX could effectively inhibit the growth and angiogenesis of Lewis lung carcinoma to the same extent as cisplatin, by suppressing the expression of HIF-1α and VEGF. Sun’s study also achieved a similar conclusion [47].

Overall, the anti-angiogenic mechanisms of GCs in cancer treatment can be summarized as follows: either (a) direct effects on tumor-derived vasculature and other cellular populations from the tumor microenvironment and (b) indirect effects via affecting cancer cell-derived factors [4]. In different subtypes of cancer, GCs regulate angiogenesis in diverse manners, including upregulation and downregulation (Table 1).

### Outstanding Questions

Though the role of GCs in the anti-angiogenic treatment of particular types of solid tumors is clear, there are other tumors for which GC treatment remains unclear. For example, in gastrointestinal cancer Busada et al. removed circulating GCs in mice by adrenalectomy, and showed the rapid onset of spontaneous gastric inflammation, oxyntic atrophy, and spasmolytic polypeptide-expressing metaplasia (SPEM), a putative precursor of gastric cancer [48]. Therefore, the authors hypothesized that endogenous glucocorticoid signaling was essential in preventing spontaneous gastric inflammation and metaplasia as well as gastric cancer development.

In renal cancer, an *in vitro* study investigated the relationship between Mife and apoptosis. Human renal carcinoma cell (RCC) lines, Caki, A498, ACHN, HT29, and SK-Hept were treated with Mife and the results revealed Mife enhanced the sensitivity of RCCs to tumor necrosis factor-related apoptosis-inducing ligand (TRAIL)-induced apoptosis through induction of DR5 expression and reduction of Bcl-2 and c-FLIP(L) expression [49]. However, the effect of Mife on TRAIL sensitization was independent of GR signaling.

In ovarian cancer, a study in 2006 demonstrated premedication with DEX prior to cisplatin or gemcitabine abrogated the growth-inhibitory or apoptotic response of the chemotherapeutic agents in the ovarian carcinoma cell lines, SKOV3, OAW-42, OVM, and M130 [50]. Furthermore, xenograft tumors in mice treated with DEX and cisplatin grew as fast in *vivo* as untreated controls, which indicated that the presence of GCs reduced the efficiency of chemotherapy. Additionally, another *in vitro* study [51] demonstrated that cytotoxic cisplatin and/or paclitaxel treatment decreased cellular attachment by 51% and resulted in significant cell death as compared to controls. But when simultaneously administered with cisplatin and/or paclitaxel treatment, DEX increased cell survival and adhesion in parallel and in a dose-dependent manner, implying DEX completely blocked apoptosis induction by cytotoxic treatment. Therefore, the use of GCs in the treatment of ovarian cancer is currently discouraged.

## Conclusions

Due to the anti-angiogenic feature of GCs, they are now regarded as an effective treatment for solid tumors. However, our review of the literature from the last ten years, reveals that the administration of GCs can be a double-edged sword in cancer therapy. Especially for breast cancer and prostate cancer, the introduction of GCs might promote angiogenesis under certain conditions. For the treatment of bladder cancer and GBM, the administration of GCs might decrease the effectiveness of radiotherapy and chemotherapy. But in melanoma, colon cancer and liver cancer, the angiostatic effect of GCs seems evident and straightforward. These discrepancies highlight the pleiotropic effects of GCs in different tumor environments. Therefore, considering the established adverse effect profile of GCs, we strongly suggest a prudent and individualized use of GCs in the treatment of solid tumors.

## Figures and Tables

**Table 1:** The effects of GCs on angiogenesis in particular solid tumors.

Cancer	GC subtype	Species	Effect on angiogenesis	Other effects	Ref
Breast cancer	TA	Rabbit	Down-regulation	-	[29]
Breast cancer	Cortisol	Mouse	Up-regulation	-	[30]
Prostate cancer	DEX	Mouse	Down-regulation	-	[31]
Prostate cancer	GC signaling pathway	Human	Up-regulation	-	[32]
Bladder cancer	DEX, PRED	Cell line	Down-regulation	-	[33]
Bladder cancer	DEX	Mouse	Down-regulation	Decrease the effectiveness of chemotherapy	[34]
GBM	DEX	Human	None	Decrease the effectiveness of radiotherapy and chemotherapy	[35]
GBM	Mife (Antagonist of GC)	Mouse	Down-regulation	-	[36]
Melanoma	DX8	Mouse	Down-regulation	-	[39]
Melanoma	PLP	Cell line	Down-regulation	-	[40]
Melanoma	HYC16	Mouse	Down-regulation	-	[41]
Colon cancer	DEX	Cell line	Down-regulation	-	[42]
Colon cancer	LCL-PLP	Mouse	Down-regulation	-	[43]
Liver cancer	Hydrocortisone	Rat	Down-regulation	-	[44]
Liver cancer	DEX	Mouse	Down-regulation	-	[45]
Lung cancer	DEX	Mouse	Down-regulation	-	[46]
Lung cancer	DEX	Mouse	Down-regulation	-	[47]

## Abbreviations

ALL: Acute Lymphoblastic Leukemia
BUD: Budesonide
CLL: Chronic Lymphoblastic Leukemia
DEX: Dexamethasone
GBM: Glioblastoma Multiforme
GC: Glucocorticoid
GR: Glucocorticoid Receptor
HL: Hodgkin’s Lymphoma
iNOS: inducible Nitric Oxide Synthase
LCL: Long-Circulating Liposome
Mife: Mifepristone
MM: Multiple Myeloma
NHL: Non-Hodgkin’s Lymphoma
NO: Nitric Oxide
PLP: Pyridoxal Phosphate
PRED: Prednisone
Rad: Radiotherapy
RCC: Renal Carcinoma Cell
SPEM: Spasmolytic Polypeptide-Expressing Metaplasia
TA: Triamcinolone Acetonide
Tmz: Temozolomide
11β: HSD 1, 2
11β: Hydroxysteroid Dehydrogenase 1, 2
